# Nodal signaling promotes vasculogenic mimicry formation in breast cancer via the Smad2/3 pathway

**DOI:** 10.18632/oncotarget.12161

**Published:** 2016-09-21

**Authors:** Wenchen Gong, Baocun Sun, Xiulan Zhao, Danfang Zhang, Junying Sun, Tieju Liu, Qiang Gu, Xueyi Dong, Fang Liu, Yong Wang, Xian Lin, Yanlei Li

**Affiliations:** ^1^ Department of Pathology, Tianjin Medical University, Tianjin, 300070, China; ^2^ Department of Pathology, Tianjin General Hospital, Tianjin Medical University, Tianjin, 300052, China; ^3^ Department of Pathology, Tianjin Cancer Hospital, Tianjin Medical University, Tianjin, 300060, China

**Keywords:** Nodal signaling, Smad2/3 pathway, VM, EMT, breast cancer

## Abstract

Vasculogenic mimicry (VM) is a nonangiogenesis-dependent pathway that promotes tumor growth and disease progression. Nodal signaling has several vital roles in both embryo development and cancer progression. However, the effects of Nodal signaling on VM formation in breast cancer and its underlying mechanisms are ill-defined. We analyzed the relationship between Nodal signaling and VM formation in one hundred human breast cancer cases and the results showed that the expression of Nodal was significantly correlated with VM formation, tumor metastasis, differentiation grade, TNM stage and poor prognosis. Furthermore, up-regulation of Nodal expression promoted VM formation of breast cancer cells *in vitro* and *in vivo*. Knockdown of Nodal expression restrained VM formation. In addition, Nodal induced EMT and up-regulated the expression of Slug, Snail and c-Myc. We found that blocking the Smad2/3 pathway by administering SB431542 inhibited VM formation in breast cancer cell lines and xenografts. Taken together, Nodal signaling through the Smad2/3 pathway up-regulated Slug, Snail and c-Myc to induce EMT, thereby promoting VM formation. Our study suggests that the Nodal signaling pathway may serve as a therapeutic target to inhibit VM formation and improve prognosis in breast cancer patients.

## INTRODUCTION

Breast cancer is the most frequently diagnosed malignant tumor among women worldwide [1]. Although the mortality rate has decreased, it still remains a leading cause of death in women. Even when breast cancer tissues have the same pathological, clinical, and hormone receptor statuses, tumors can still have different metastatic potentials. The tumor microenvironment and vascular network formation may play important roles in these differences [2]. Moreover, the tumor microenvironment facilitating their invasion, dissemination and metastasis had been arisen concerned recently [3, 4]. The blood supply supporting the growth of tumors facilitates cancer progression by allowing tumor cells to travel to distant sites. Nevertheless, angiogenesis is not the only process by which tumors acquire their blood supply.

Vasculogenic mimicry (VM) was reported as a nonangiogenesis-dependent pathway in 1999 [5]. Aggressive cancer cells were shown to form vascular networks by themselves without the involvement of endothelial cells. VM can feed the growing tumor and promote disease progression [6, 7]. Moreover, the presence of VM has been reported in many aggressive tumors, such as melanoma, prostate carcinoma, ovarian carcinoma, hepatocellular carcinoma, and lung cancer [6, 8–11]. Our previous studies showed that the presence of VM in breast cancer was associated with metastasis and poor prognosis [12–14]. Recently, it was confirmed that VM serves as a driver of metastasis in a breast cancer model [15].

Nodal is a member of the TGF-β superfamily and has several critical roles in embryo development. It is normally expressed during embryogenesis, and promotes mesendoderm specification and left-right asymmetry [16–18]. However, its re-expression induces increased aggressiveness and tumorigenicity in cancer cells in melanoma, glioma, and prostate cancer [16, 19–21]. Nodal predominantly binds to activin-like kinase type II (ActRIIB) and type I (ALK4/7) receptors, which leads to phosphorylation of ALK4/7. Activation of the receptors promotes intracellular phosphorylation of Smad2/3, which then interacts with SMAD4, followed by translocation to the nucleus, thereby regulating target genes [22]. Nodal expression is correlated with tumor progression, poor prognosis and angiogenesis [16, 23–25]. Moreover, recent studies have shown that Nodal may regulate breast cancer progression and metastasis [24, 26]. However, the underlying mechanism of Nodal promotion of breast cancer development remains to be characterized. Additionally, whether Nodal signaling regulates VM formation and its effects on breast cancer are ill-defined.

In this study, we focused on determining the function of Nodal in VM formation and the role of the Smad2/3 pathway in this process. Here, we demonstrate that Nodal overexpression was correlated with poor prognosis in patients and the presence of VM in human breast cancer. We found that Nodal signaling regulated VM formation and induced epithelial-mesenchymal transition (EMT) *in vitro* and *in vivo* principally via the Smad2/3 pathway. Furthermore, the application of SB431542 in a mouse model of breast cancer inhibited VM formation in xenografts. Thus, Nodal might serve as a therapeutic target for inhibiting VM formation and improving the prognosis in breast cancer.

## RESULTS

### Nodal expression correlates with breast cancer progression

**Figure 1:**
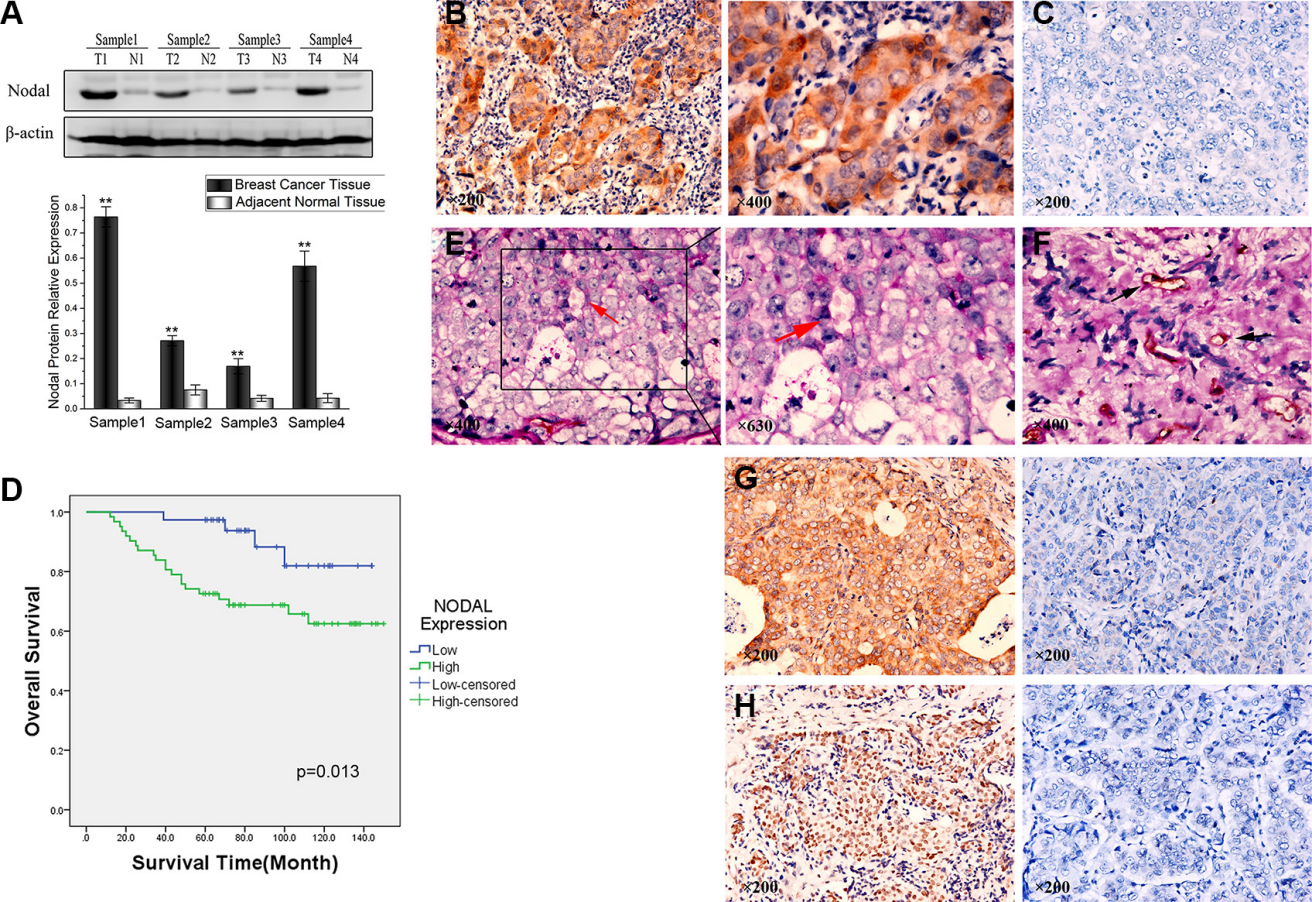
Expression of Nodal correlates with vasculogenic mimicry (VM) and poor prognosis in human breast cancer samples (**A**) Nodal expression levels in four pairs of human breast cancer tissues and matched adjacent non-tumorous tissues were evaluated by Western blot analysis. The chart shows the relative expression of Nodal in the breast cancer tissues and matched adjacent non-tumorous tissues. (**B**) Breast cancer specimens were analyzed by immunohistochemistry. Nodal was predominantly localized in the cytoplasm of cancer cells (magnification, 200× and 400×). (**C**) Negative expression of Nodal in breast cancer specimens (magnification, 200×). (**D**) Overall survival of patients with Nodal-positive and Nodal-negative samples. Kaplan–Meier analysis showed that the patients with Nodal-positive samples displayed poorer prognosis (χ^2^ = 6.206, *p* = 0.013 determined with a log-rank test). (**E**) CD31/PAS double staining displayed VM channels in breast cancer specimens. The channels (red arrowhead) lined with tumor cells contained red blood cells and were CD31-negative and PAS-positive (magnification, 400× and 630×). (**F**) The blood vessels were CD31-positive (black arrowhead) (magnification, 400×). (**G**) Positive VE-cadherin expression in breast cancer specimens and negative VE-cadherin expression for comparison (magnification, 200×). (H) Positive Slug expression in breast cancer specimens and negative Slug expression for comparison (magnification, 200×). **p < 0.01.

To examine Nodal expression in human breast tissue, we compared Nodal protein levels in breast cancer samples and paired adjacent normal tissues from patients. The Western blot results showed that Nodal protein levels were significantly up-regulated in breast cancer tissues compared with adjacent normal tissues (Figure 1A). To further investigate Nodal expression, breast cancer tissue samples from 100 patients were analyzed by immunohistochemistry. As shown in Figure 1B, Nodal was predominantly localized in the cytoplasm of cancer cells (the negative staining of Nodal is shown in Figure 1C for comparison). Under high-power magnification, 10 random fields from each specimen were selected, and > 500 cells were assessed to determine the percentage of positive cells. Percentages ≥ 10% were considered positive samples. IHC of 100 cases showed that 62 tumors had strong Nodal expression, and the other 38 tumors had relatively weak Nodal expression.

As it shown in Table 1, 59.7% (37/62) of cases with Nodal overexpression (Nodal^high^) underwent lymph node metastasis compared with 21.1% (8/38) of cases with low Nodal expression (Nodal^low^) (*p* = 0.000). Moreover, 38.7% (24/62) of the Nodal^high^ group and 10.5% (4/38) of the Nodal^low^ group were diagnosed as differentiation grade III (*p* = 0.003). Similarly, TNM clinical stages of cases in the Nodal^high^ and Nodal^low^ groups showed significant differences (*p* = 0.045). Finally, Kaplan–Meier survival analysis indicated that the Nodal^high^ group had poor overall survival compared with the Nodal^low^ group (*p* = 0.013, Figure 1D). Therefore, we concluded that the expression of Nodal was significantly correlated with tumor metastasis, differentiation grade, TNM stage and poor prognosis but not age and tumor size.

**Table 1:** Correlation between Nodal expression and clinicopathologic parameters, VM formation, VE-cadherin and Slug expression in breast cancer

Factors	Nodal expression		*P*
	+ (%)	– (%)	
**Age (years)**			0.532
**< 50**	39 (62.9)	22 (57.9)	
**≥ 50**	23 (37.1)	16 (42.1)	
**Tumor size (diamater)**			0.512
**D ≤ 2**	17 (27.4)	12 (31.6)	
**2 < D ≤ 5**	41 (66.1)	26 (68.4)	
**D > 5**	4 (6.5)	0	
**Nodal status**			0.000*
**Negative**	25 (40.3)	30 (78.9)	
**Positive**	37 (59.7)	8 (21.1)	
**Differentiation grade**			0.003*
**I/II**	38 (61.3)	34 (89.5)	
**III**	24 (38.7)	4 (10.5)	
**Tumor stage**			0.045*
**I**	5 (8)	9 (23.7)	
**II**	40 (64.5)	25 (65.8)	
**III**	13 (21)	4 (10.5)	
**IV**	4 (6.5)	0	
**VM**			0.005*
**No**	42 (67.7)	35 (92.1)	
**Yes**	20 (32.3)	3 (7.9)	
**VE-cadherin**			0.000*
**–**	17 (27.4)	24 (63.2)	
**+**	45 (72.6)	14 (36.8)	
**Slug**			0.001*
**–**	12 (19.4)	19 (50)	
**+**	50 (80.6)	19 (50)	

* Significantly different.

### Expression of Nodal is associated with the presence of VM in breast cancer tissues

In addition, CD31/PAS double staining was used to identify VM in tumors, which has been performed in many studies [27–29]. Among the 100 samples of breast cancer tissue, 23 samples showed the formation of vascular-like networks that were CD31-negative, PAS-positive and contained red blood cells (Figure 1E, red arrowhead). Compared with VM, the vessels formed by endothelial cells were identified by CD31 staining (Figure 1F, black arrowhead). The results showed that 32.2% (20/62) of the Nodal^high^ group displayed VM, while in the Nodal^low^ group, only 7.9% (3/38) had VM (*p* = 0.005). Consequently, expression of Nodal was found to be positively associated with the presence of VM.

Moreover, we found that Nodal was also associated with the expression of the endothelial-specific marker VE-cadherin. We found that 72.6% (45/62) of the Nodal^high^ group overexpressed VE-cadherin (Figure 1G) compared with 36.8% (14/38) of the Nodal^low^ group (*p* = 0.000). Compared with the expression of Slug in the Nodal^low^ group, 80.6% (50/62) of the cases in the Nodal^high^ group were identified as Slug-positive (*p* = 0.001) (Figure 1H). Based on these data, we concluded that Nodal was correlated with VM formation, VE-cadherin and Slug expression.

### Expression of Nodal in breast cancer cell lines, and Nodal signaling activates the Smad2/3 pathway

**Figure 2:**
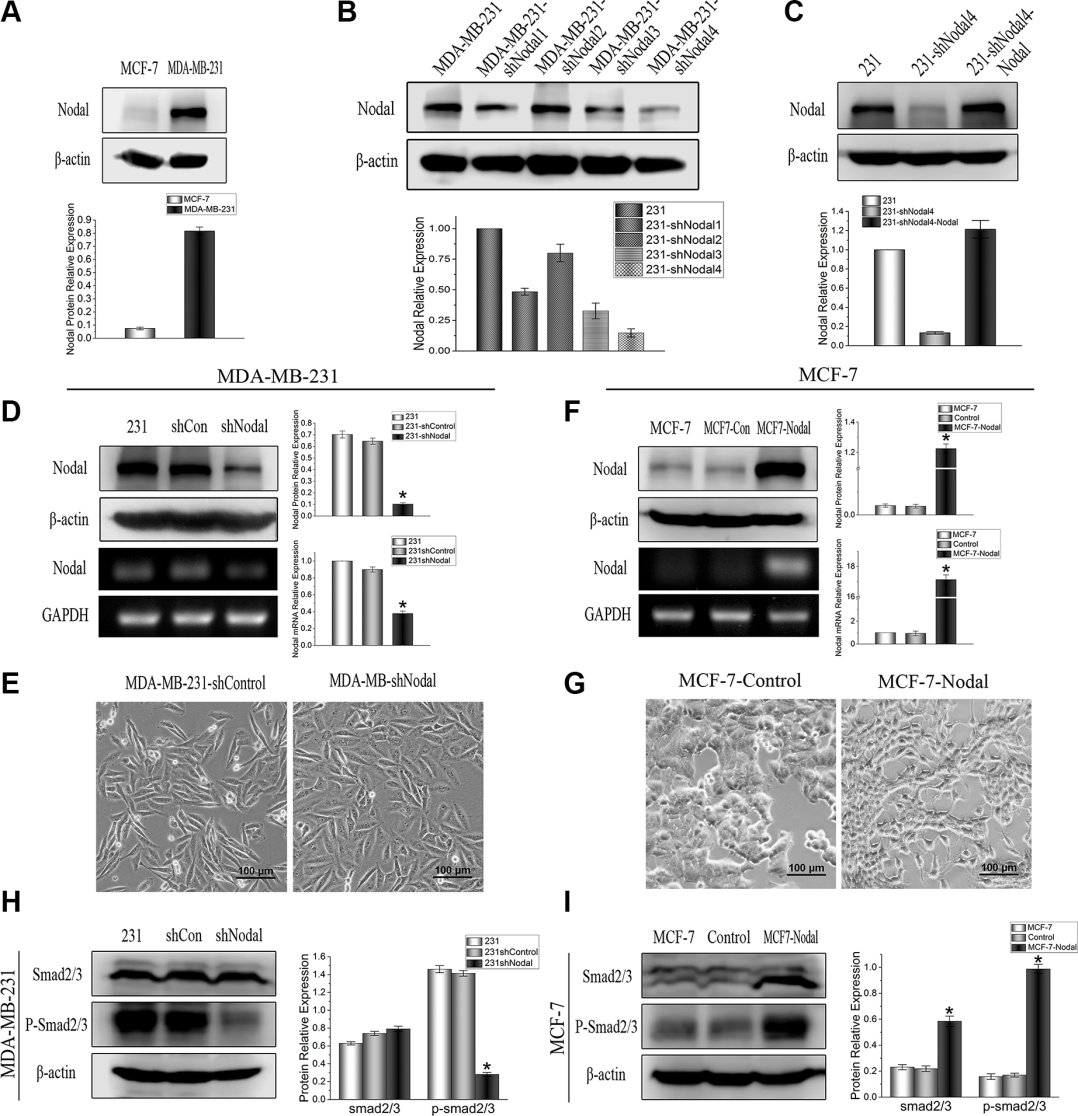
Expression of Nodal in breast cancer cell lines and establishment of stable Nodal knockdown or Nodal-overexpressing cell lines (**A**) The basic expression of Nodal protein in breast cancer cell lines MCF-7 and MDA-MB-231. (**B**) MDA-MB-231 cells were stably transfected with 4 shRNAs, and their effects were examined by western bolt analysis. (**C**) The rescue experiments were performed by overexpressing Nodal in Nodal knockdown shNodal4 cells. The Nodal expression in 231-shNodal4 had got recover. (**D**) MDA-MB-231 cells were stably transfected with shNodal and shControl vectors, and efficiencies were confirmed by Western blot and RT-PCR analyses. (**E**) Morphological changes in MDA-MB-231-shNodal cells were compared with shControl cells. (**F**) MCF-7 cells were stably transfected with Nodal cDNA and control vectors, and efficiencies were confirmed by Western blot and RT-PCR analyses. (**G**) Morphological changes in MCF-7-Nodal cells were compared with control cells. (**H**) (**I**) The expression of Smad and p-Smad proteins was evaluated by Western blot analysis in the indicated cells. Assays were performed in triplicate. The data are presented as the mean ± standard deviation (SD).*p < 0.05.

To identify the role and mechanism of Nodal in breast cancer, the breast cancer cell lines MCF-7 and MDA-MB-231 were selected as *in vitro* models. The expression levels of Nodal were assessed by Western blot analysis, and the results showed that MCF-7 cells had low-level Nodal expression, while MDA-MB-231 cells presented high levels (Figure 2A). To establish stable Nodal knockdown or Nodal-overexpressing cells, MDA-MB-231 cells were infected with four lentiviral vectors expressing Nodal shRNA or a non-target shRNA control lentiviral vector. Their effects were examined by western bolt (Figure 2B). Among the four shRNAs, shNodal4 most efficiently knocked down Nodal expression by more than 80% (Figure 2B), thus the shNodal4 was chosen to use in the followed functional experiments (Figure 2D). In addition, the rescue experiments had been performed by overexpressing Nodal in Nodal knockdown shNodal4 cells. The western bolts were performed (Figure 2C), and the Nodal expression in 231-shNodal4 had got recover. To exclude the off target effect the functional experiments were also performed and the results were shown in Supplementary Figure S2. MCF-7 cells were infected with a lentiviral vector overexpressing a Nodal cDNA or a control lentiviral vector. The transfection efficiencies in these cells were confirmed via Western blot and RT-PCR analysis (Figure 2F). Compared with the MDA-MB-231-shControl cells, there was no significant reversal of the EMT phenotype in the MDA-MB-231-shNodal cells (Figure 2E); however, Nodal overexpression resulted in alterations in MCF-7 cells from epithelial to fibroblast-like morphologies (Figure 2G).

Similar to other members of the TGF-β superfamily, Nodal binds to activin-like kinase type II and type I receptors, which leads to phosphorylation of ALK4/7. Activation of the receptors phosphorylates Smad2/3 and regulates target genes [22]. Therefore, phosphorylated Smad2/3 levels were measured in stable Nodal knockdown or Nodal-overexpressing breast cancer cells to determine the effect of Nodal on the Smad2/3 pathway. The Western blot results showed that the knockdown of Nodal in MDA-MB-231 cells decreased Smad2/3 phosphorylation (Figure 2H), and overexpression of Nodal in MCF-7 cells dramatically increased p-Smad2/3 levels (Figure 2I). Additionally, the expression of Smad3 was also increased in MCF-7-Nodal cells. To determine whether the Smad2/3 pathway is essential for Nodal signaling in breast cancer, a specific molecular inhibitor, SB431542 (SB), that inhibits the activin type I receptor was used in further experiments.

### Nodal signaling promotes the formation of VM-like channels, and this process can be inhibited by SB431542 *in vitro*

**Figure 3:**
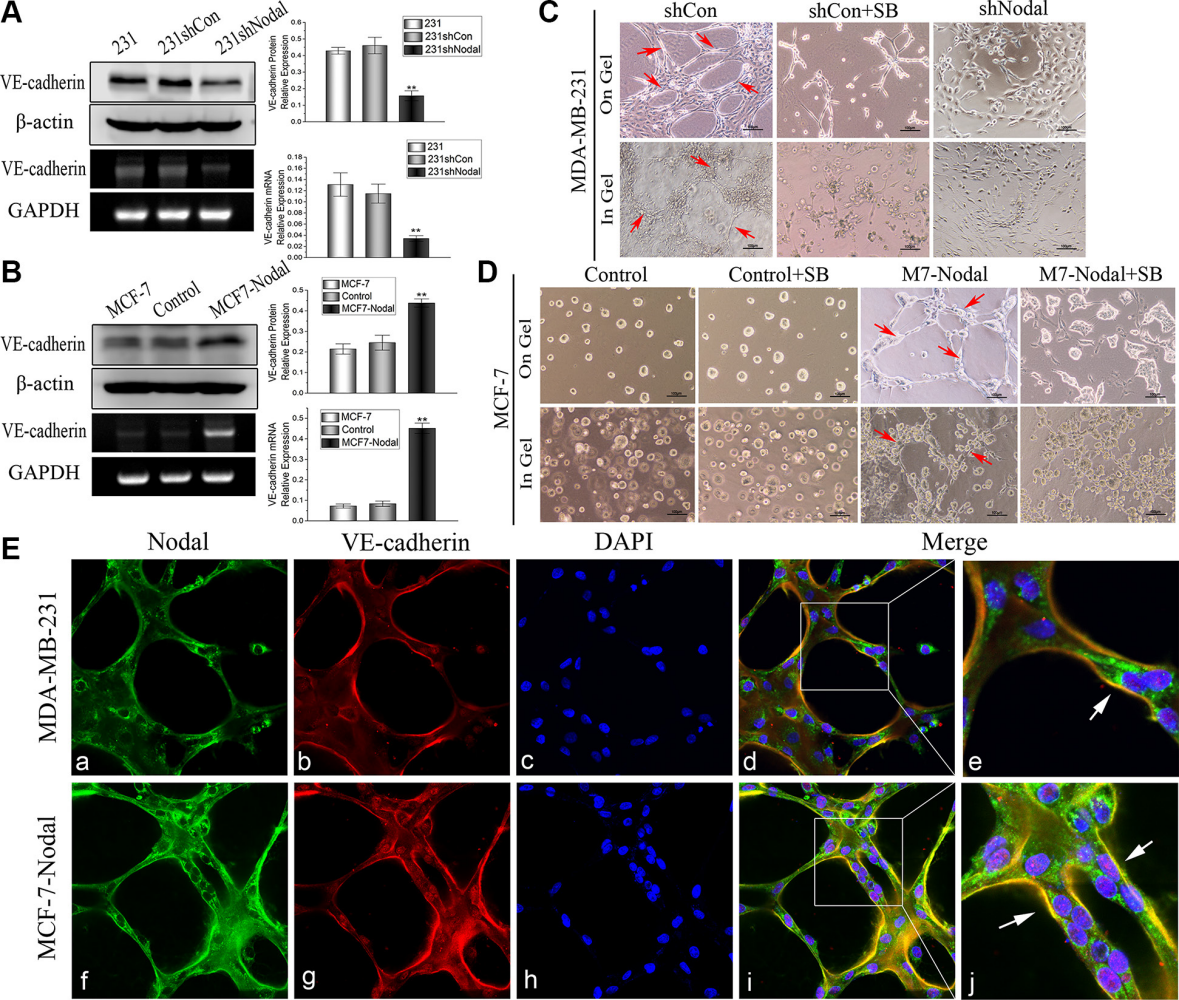
Nodal signaling promotes VM formation, and the effect of Nodal is inhibited by SB *in vitro* (**A**) (**B**) The expression of VE-cadherin proteins was evaluated by Western blot and RT-PCR analyses in the indicated cells. Assays were performed in triplicate. The data are presented as the mean ± standard deviation (SD) **p < 0.01 (**C**) MDA-MB-231 cells did not form VM channels when Nodal expression was knocked down or when they were treated with SB (10 μM) on Matrigel and in Matrigel. MDA-MB-231-shControl cells formed typical VM channels (red arrowhead). (**D**) MCF-7 cells cannot form VM channels, but up-regulating Nodal expression led to the formation of VM channels both on Matrigel and in Matrigel (red arrowhead). Meanwhile, SB could neutralize the effect of Nodal signaling on VM formation. Scale bar = 50 μm. (**E**) The VM channels formed by MDA-MB-231 cells or MCF-7-Nodal cells were assessed by immunofluorescence and confocal microscopy. a, f Nodal staining in the VM channels was concentrated in the cytoplasm and the outer edges of the channels (magnification, 200×). (b, g) VE-cadherin staining of the VM channel was principally concentrated in the wall of the channel (magnification, 200×). (c, h) The nuclei were stained by DAPI (magnification, 200×). (d, e, i, j) The merge pattern showed that Nodal signaling was associated with the expression of VE-cadherin in VM networks. Nodal expression was concentrated in the cells that formed the VM networks and in the outer edges of the structures overexpressing VE-cadherin in the walls (magnification, 200× and 500×).

Because Nodal was correlated with VM formation, we further examined the role of Nodal in VM formation *in vitro*. VE-cadherin is an endothelial-specific marker expressed in many highly aggressive tumor cells, and it has also been linked to VM [30]. Therefore, Western blot and RT-PCR analyses were performed. We found that decreased expression of Nodal in MDA-MB-231 cells down-regulated the VE-cadherin protein and mRNA expression (Figure 3A). Nodal overexpression up-regulated VE-cadherin protein and mRNA levels (Figure 3B). Furthermore, on Matrigel and in Matrigel 3D cultures as well-established VM formation *in vitro* model were investigated. As shown in Figure 3C, 231-shControl cells formed typicalchannel-like structures on Matrigel and in Matrigel 3D cultures (red arrowhead), while there were no integrated VM-like channels in 231-shNodal cells. Meanwhile, the ability of 231-shControl cells to form these structures could be inhibited by SB431542. In addition, the MCF-7-Control cells did not form tubular structures, but Nodal overexpression promoted the formation of channel-like networks both on Matrigel and in Matrigel as shown in MCF-7-Nodal cells (Figure 3D, red arrowhead). Similarly, SB431542 also could neutralize the effect of Nodal on VM formation (Figure 3D).

To investigate the features of the channel-like structures and the relationship between Nodal signaling and VM, the VM-like networks on Matrigel were assessed with immunofluorescence and analyzed by confocal microscopy. The staining of pipe-like structures that formed by 231 cells and MCF7-Nodal cells showed that these cells overexpressed Nodal (Figure 3E a, f). Simultaneously, channel-like structures overexpressed the endothelial-specific marker VE-cadherin, especially on the wall of these structures (Figure 3E b, g). Interestingly, as shown in (Figure 3E e and 3E j), Nodal was concentrated in the cells that formed the VM networks and in the outer edges of these structures. At same time, these VM channel walls overexpressed VE-cadherin (Figure 3 E e, j white arrowhead). Therefore, Nodal promoted VM formation *in vitro* and was associated with the expression of VE-cadherin in VM structures.

### Nodal signaling up-regulates VM-associated protein expression via the Smad2/3 pathway

**Figure 4:**
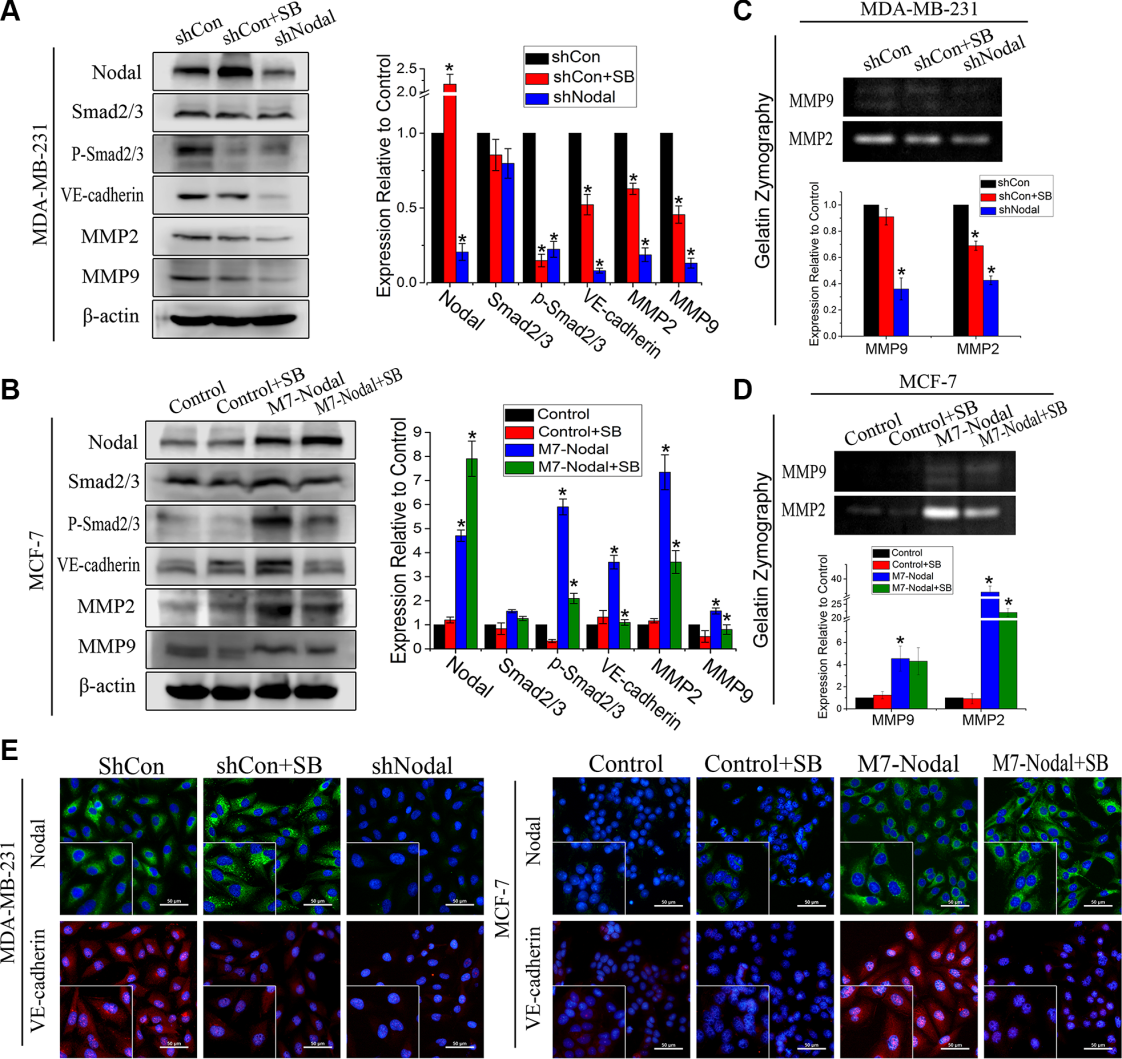
Nodal signaling via the Smad2/3 pathway up-regulated VM-associated protein expression (**A**, **B**) The expression of Nodal, Smad2/3, p-Smad2/3, VE-cadherin, MMP2, and MMP9 was evaluated by Western blot analyses in the indicated cells (including SB-treated groups). (**C**, **D**) The activities of MMP2 and MMP9 were evaluated by gelatin zymography. (**E**) Immunofluorescences staining was performed to verify the expression of Nodal and VE-cadherin in the indicated cells on glass slides. Assays were performed in triplicate. The data are presented as the mean ± standard deviation (SD).*p < 0.05, scale bar = 50 μm.

To verify the effects of SB431542 on VM formation, the expression levels of Nodal, p-Smad2/3 and VE-cadherin were determined in MCF-7, MCF-Nodal and MDA-MB-231 cells following treatment with SB. Smad2/3 phosphorylation levels were dramatically inhibited. Interestingly, we observed that in these cells, the level of Nodal expression increased when they were treated with SB (Figure 4A, 4B). At the same time, the expression of VE-cadherin was down-regulated by SB431542 (Figure 4A, 4B). The results were also verified by immunofluorescence staining (Figure 4E). MMP-2 and MMP-9 are important members of the MMP family that play crucial roles in cell plasticity and VM formation [6, 10]. MMPs can be produced by the cancer cells in VM networks, resulting in extracellular matrix remodeling and promotion of VM formation. To determine the effect of Nodal on MMP-2 and MMP-9, gelatin zymography and Western blot analyses were performed. Compared with 231-shControl cells, knockdown of Nodal reduced the expression and activities of MMP2 and MMP9 (Figure 4A, 4C). Additionally, overexpression of Nodal also resulted in a significant increase in the expression and activities of MMP2 and MMP9 (Figure 4B, 4D). Furthermore, we observed that MCF-7-Con, MCF-7-Nodal and MDA-MB-231-shCon cells treated with SB431542 also showed decreased expressions and activities of MMP2 and MMP9 to some extent. The results indicated that Nodal was essential to VM formation *in vitro*. Nodal signaling may involve complicated pathways, but the Smad2/3 signaling pathway plays an important role in the formation of VM.

### Nodal facilitates migration and invasion in breast cancer cells

**Figure 5:**
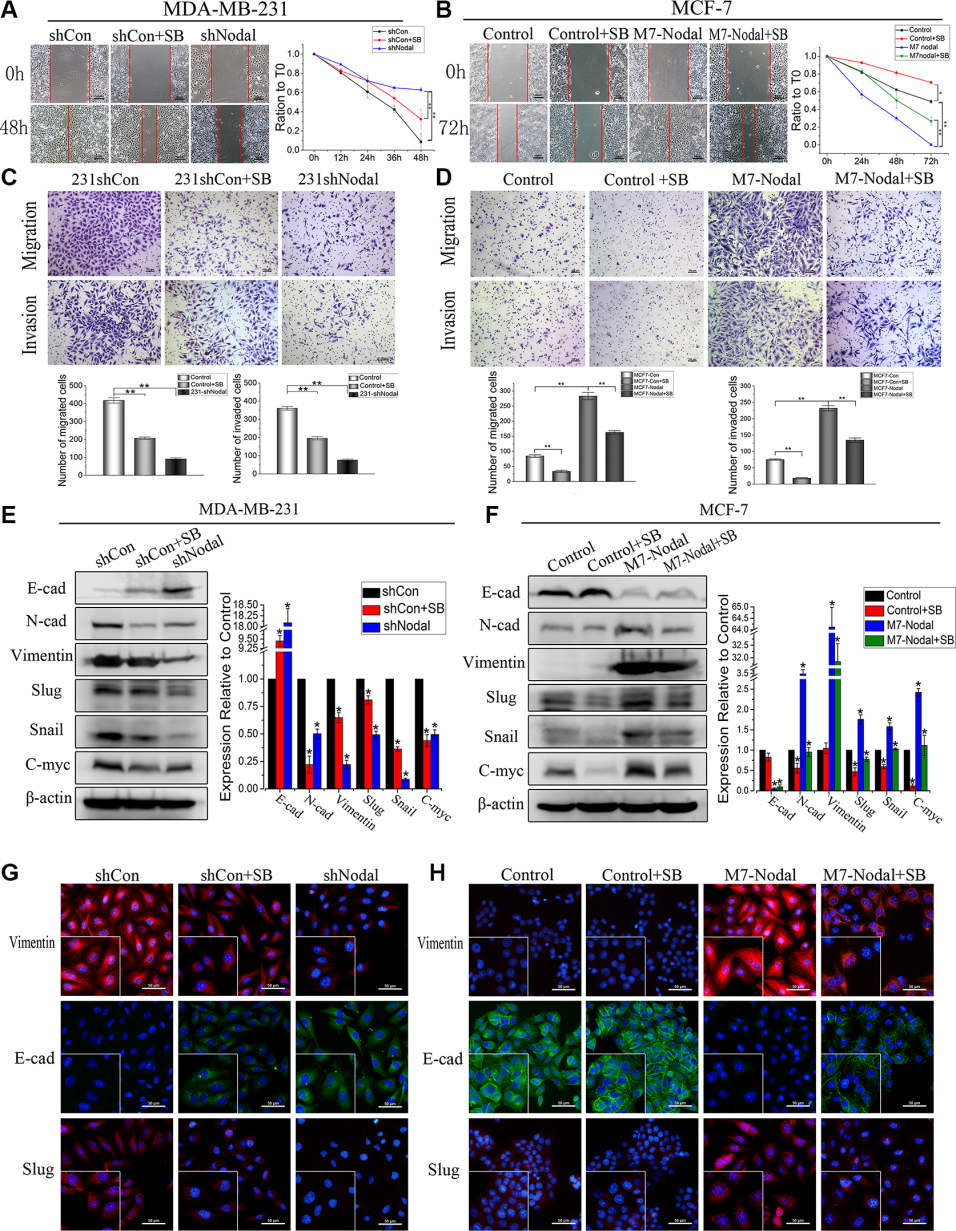
Nodal induces EMT via the Smad2/3 pathway in breast cancer cells MDA-MB-231-shControl cells, MDA-MB-231-shNodal cells, MCF-7-Control cells, MCF-7-Nodal cells and SB treatment groups were evaluated. (**A**, **B**) Wound-healing assays were performed in the indicated groups. Scale bar = 200 μm. (**C**, **D**) Transwell assays were performed in the indicated groups. Scale bar = 100 μm (**E**, **F**) Western blot analysis showed the protein expression of E-cadherin, N-cadherin, vimentin, Slug, Snail and c-Myc in the indicated cells. (**G**, **H**) Immunofluorescence analysis was performed to verify the expression of vimentin, E-cadherin and Slug in the indicated cells. Assays were performed in triplicate. The data are presented as the mean ± standard deviation (SD). *p < 0.05, **p < 0.01, scale bar = 50 μm.

VM formation involves tumor cell-mediated simulation of endothelial cells. EMT contributes to tumor cell plasticity and the acquisition of mesenchymal properties. Thus, EMT has been proposed as a critical process in VM formation [6, 31]. EMT and VM formation were associated with cell migration and invasion; therefore, wound-healing assays and transwell assays were performed to investigate the effects of Nodal in breast cancer cells. In wound-healing assays, knockdown of Nodal expression decreased the migratory activities of MDA-MB-231 cells, and treatment with SB inhibited migration (Figure 5A, *p* < 0.01). Increased Nodal expression significantly promoted the migration of MCF-7 cells, but this effect was compromised by SB (Figure 5B, *p* < 0.01). Furthermore, transwell assays were used to evaluate cell migration and invasion. The results suggested that compared with MDA-MB-231-shCon, 231-shNodal cells had reduced migration and invasion (Figure 5C, *p* < 0.01). Additionally, significant differences were found between MCF-7-Con and MCF-7-Nodal cells (Figure 5D, *p* < 0.01). However, we found that the significantly increased migration and invasion were limited by the inhibitor SB in MCF-7-Nodal cells, and SB also neutralized the migration and invasion of MDA-MB-231-shCon cells (Figure 5C, D, *p* < 0.01).

### Nodal signaling enhances the levels of EMT markers and up-regulates the expression of Slug, Snail and c-Myc

There are many common pathways shared by EMT and VM formation. To further identify the underlying mechanisms of Nodal on VM formation, EMT-associated markers were evaluated. The results showed that compared with MCF-7-Con cells, increased expression of Nodal up-regulated the mesenchymal markers N-cadherin and vimentin and down-regulated the epithelial marker E-cadherin. Notably, MCF-7 cells overexpressing Nodal had increased levels of the transcription factors Snail, Slug and c-Myc. In contrast, knocking down Nodal expression led to contrary results of these genes in 231-shNodal cells compared with MDA-MB-231-shCon cells. Moreover the epithelial marker E-cadherin was up-regulated, and N-cadherin, vimentin, Snail, Slug and c-Myc were down-regulated to different degrees when MCF-7-Con, MCF-7-Nodal and MDA-MB-231-shCon were treated with SB (Figure 5E, 5F). Using immunofluorescence, we further verified these results and showed that Nodal, which is expressed in the cytoplasm, induced EMT and up-regulated the expression of the transcription factor Slug. The effects of Nodal could be neutralized by SB to some extent.

In general, these data showed that Nodal signaling could induce EMT and up-regulate the expression of Slug, Snail and c-Myc via the Smad2/3 pathway.

### Nodal signaling promotes tumorigenicity and VM formation in breast cancer *in vivo*, and SB431542 inhibits its effects in a mouse model

**Figure 6:**
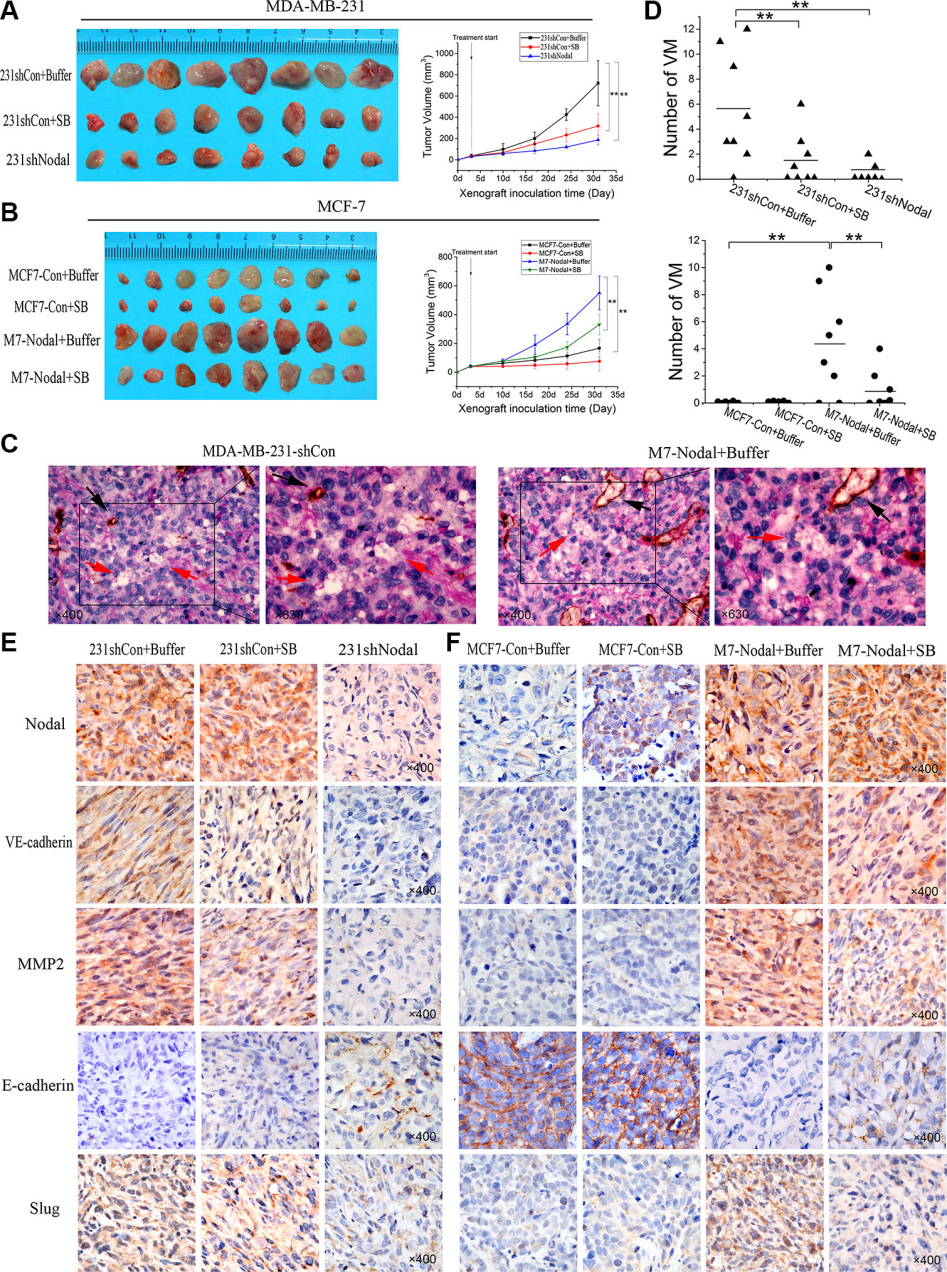
Nodal signaling promotes tumorigenicity and VM formation in breast cancer xenografts, and application of SB inhibits its function *in vivo* (**A**, **B**) MDA-MB-231-shControl, MDA-MB-231-shNodal, MCF-7-Control and MCF-7-Nodal cells were subcutaneously injected into BALB/c-nu/nu mice. The SB solution (10 mg/kg/mouse) was intraperitoneally injected in the treatment groups on alternate days. The data are presented as the mean ± SD. (**C**) CD31/PAS double staining displayed VM channels in xenografts. The channels (red arrowhead) lined with tumor cells contained red blood cells and were CD31-negative and PAS-positive. The blood vessels were CD31-positive in comparison (black arrowhead). (**D**) Quantification of VM observed in the MDA-MB-231-shControl group and the MCF-7-Nodal group. **p < 0.01. (**E**, **F**) The expression of Nodal, VE-cadherin, vimentin (Vim), MMP2, E-cadherin and Slug was evaluated by IHC staining in the indicated groups (magnification, 400×).

To validate the function of Nodal signaling and to investigate the feasibility of blocking the Smad2/3 pathway to reduce VM formation *in vivo*, 231-shCon, 231-shNodal, MCF-7-Con and MCF-7-Nodal cells were subcutaneously injected into BALB/c-nu/nu mice. Three days after inoculation, SB solution (10 mg/kg/mouse) was intraperitoneally injected in the treatment groups on alternate days, and the control groups were treated with a placebo. Compared with the 231-shCon group, the tumors grew at a significantly slower rate in the 231-shNodal group (*p* < 0.01, Figure 6A b). The volume of the tumors in mice in the 231-shNodal group was significantly reduced (Figure 6A a). Meanwhile, Nodal signaling significantly promoted tumor growth in the M7-Nodal group compared with the control group (*p* < 0.01, Figure 6B). Moreover, when the 231-shCon, MCF7-Nodal and MCF-7-Con groups were treated with SB431542, the tumor growth rate was inhibited, and tumorigenicity was weakened (Figure 6A, 6B).

To verify the effect of Nodal signaling in VM formation *in vivo*, immunohistochemical (IHC) staining was performed in xenografts. Endomucin/periodic acid–Schiff (PAS) double staining assays showed that Nodal knockdown strongly inhibited the formation of VM channels in the 231-shNodal group (2/8) compared to the 231-shCon group (7/8) (Figure 6C). Additionally, treatment with SB also decreased the number of VM channels *in vivo* (4/8) (Figure 6D, *p* < 0.01). Furthermore, VM formation was negative in the MCF-7-Con and MCF-7-Con SB treated group (0/8). Consistent with the *in vitro* assays, up-regulated Nodal signaling dramatically increased the number of VM channels in xenografts (6/8) (Figure 6D, *p* < 0.01). However, treatment with SB could efficiently inhibit this effect on VM formation (3/8, *p* < 0.01).

In addition, we further measured the expression of Nodal and the VM marker VE-cadherin in tumor tissues. The expression of Nodal was down-regulated in the 231-shNodal group and up-regulated in the M7-Nodal group compared with their respective controls. VE-cadherin expression was consistent with the results of VM formation in xenografts. Nodal expression was positively correlated with VE-cadherin. Moreover, compared with the control group, the MCF-7-Nodal group displayed lower expression of the epithelial marker E-cadherin and higher levels of MMP2 and Slug (Figure 6F), but the 231-shNodal group had contrasting results compared with the 231-shCon group (Figure 6E). Notably, in the SB-treated groups (231-shCon+SB and M7-Nodal+SB), E-cadherin was up-regulated, and VE-cadherin, MMP2 and Slug were down-regulated compared with the buffer-treated groups. However, there were nuance differences between the MCF-7-Con and MCF-7-Con+SB groups (Figure 6E, 6F).

In sum, these results indicate that Nodal signaling induces EMT and promotes VM formation *in vivo*. SB431542 reduced VM formation in xenografts.

## DISCUSSION

In the present study, we investigated the role of Nodal and demonstrated for the first time that Nodal signaling facilitated VM formation in breast cancer. We found that Nodal signaling up-regulated Slug, Snail and c-Myc via the Smad2/3 pathway, resulting in the induction of EMT, thereby promoting VM formation. Treatment with SB431542 suppressed the formation of VM in breast cancer *in vitro* and *in vivo*. Thus, these data indicate that Nodal signaling not only was identified as a prognostic marker but also may serve as a therapeutic target.

Nodal is an embryonic morphogen that promotes the displacement of the anterior visceral endoderm and determines the left-right asymmetry [32]. However, recent studies showed that its re-expression in tumors increased cancer cell aggressiveness and tumorigenicity [33–36]. A previous study showed that Nodal signaling played a key role in melanoma cell plasticity and tumorigenicity [34]. In human melanoma samples, Nodal expression was highly correlated with metastasis [37]. Nodal has also been shown to promote progression and stem cell-like phenotypes in pancreatic cancer [38, 39]. Moreover, in breast cancer patients, Nodal was positively associated with tumor stage, lymph node status and tumor grade [26]. Consistent with previous studies, we found that Nodal was a marker for metastasis and poor prognosis in breast cancer. Nodal expression in human breast cancer specimens was significantly correlated with tumor metastasis, differentiation grade and TNM stage. Here, we show that Nodal expression was strongly linked to VM in breast cancer samples. Moreover, Nodal signaling was also associated with the expression of VE-cadherin and Slug.

Vasculogenic mimicry has been reported in many malignant tumor types [8–13]. VM was associated with high tumor grade and more aggressive, poorly differentiated, and highly metastatic tumors [28, 40]. Our previous study demonstrated that VM is connected with endothelium-dependent vessels using mouse models [41]. We injected activated carbon particles into the tail veins of mice and observed a number of activated carbon particles in the VM and the endothelium-dependent vessels. Therefore, on the one hand, VM can feed the growing tumor by providing a blood supply, but on the other hand, it provides an escape route for metastatic cells via the leaky pipe-like structure of VM [5, 42]. Prior to angiogenesis, VM initially sustains tumor growth and provides space for endothelial cell growth. In addition, our previous studies have shown that VM was related to poor prognosis, progression and metastasis in breast cancer [12–14]. A recent report published in *Nature* directly verified that VM drives tumor cell metastasis to distant regions in a breast cancer model [15]. Consequently, restraining VM formation is essential to improve the prognosis of breast cancer patients. In the current study, we demonstrated that Nodal plays an essential role in promoting VM formation in breast cancer cells. Using an *in vitro* 3D Matrigel culture, Nodal up-regulation in MCF-7 cells, which do not form VM channels, was shown to facilitate the formation of channel-like structures both on Matrigel and in Matrigel. Moreover, knocking down the Nodal expression in MDA-MB-231 cells inhibited their ability to form the VM channels. Moreover, we demonstrated that Nodal promoted VM marker VE-cadherin and MMP2 and MMP9 expression, which serve as the major mediators of VM [43, 44]. In addition, a previous study demonstrated that Nodal signaling also promoted endothelial vessel recruitment [24]. Our previous study described the “three stages of tumor microcirculation” in melanomas [7]. The three stages include VM channels, mosaic blood vessels and endothelial vessels, which compose the tumor microenvironment [45, 46]. Therefore, we inferred that Nodal might play vital roles in different stages of microcirculation, at least in VM formation and endothelial vessel formation. Further studies are needed to verify this hypothesis.

VM formation involves tumor cells that have acquired mesenchymal properties. EMT is the process by which epithelial cells acquire the characteristics and functions of mesenchymal cells [47, 48]. Our group has proposed that EMT plays an important role in facilitating VM formation [6, 31]. Previous studies have shown that Nodal induces EMT, promoting the aggressive phenotype [49, 50]. In this study, to further understand the effect of Nodal on VM formation, EMT-associated markers were evaluated, and functional assays were conducted. We found that up-regulated Nodal induced MCF-7 to acquire a fibroblast-like morphology, while the E-cadherin level decreased, and expression of the mesenchymal markers N-cadherin and vimentin significantly increased. Moreover, wound-healing assays and transwell assays verified that Nodal signaling promoted migration and invasion of breast cancer cells. In contrast, knocking down the Nodal expression of MDA-MB-231 cells had the reverse effect on the expression of EMT markers. Consistent with the Western blot results, the migration and invasion of MDA-MB-231 cells were inhibited by knocking down Nodal expression. Accordingly, Nodal signaling induces EMT in breast cancer, thereby contributing to VM formation.

Nodal binds type I (ALK4/7) and type II (ActRIIB) activin-like kinase receptors, resulting in phosphorylation of Smad2/3, which interacts with SMAD4 and translocates to the nucleus, thereby regulating target genes [32]. Recently, a non-SMAD pathway has been identified as well, showing that Nodal induces ERK1/2 activation, which was mediated by ALK4/7 activity [50]. However, whether the Smad2/3 pathway was involved the VM formation was unclear. In the current study, we demonstrate that the Smad2/3 pathway plays an important role in Nodal promotion of VM formation. Blocking the Smad2/3 pathway restrained the formation of VM. And we illustrated Nodal signaling and effect of SB431542 in Supplementary Figure S1. We demonstrated that in an *in vitro* 3D Matrigel culture, SB431542 inhibited the formation of VM both in MDA-MB-231-shCon cells and MCF-7-Nodal cells. To verify the effect of SB431542, Western blots were performed and showed that phosphorylation of Smad2/3 was inhibited, and expression of VE-cadherin, MMP2 and MMP9 was reduced. However, it was noteworthy that Nodal expression increased when the Smad2/3 pathway was inhibited. We hypothesized that there may be a negative feedback loop in the Nodal/ALK4/7 pathway. Another possibility is that a non-Smad compensatory pathway may increase, promoting Nodal expression. Further studies are needed to investigate this hypothesis. That using genomic microarray technology to probe the differences after SB431542 treatment and explore its effects. Furthermore, blocking the Smad2/3 pathway also decreased the expression of EMT-associated markers. In addition, previous studies have shown that that Nodal signaling post-translationally regulates the c-Myc and p27 proteins [51]. Consistent with previous studies, our findings showed that Nodal signaling up-regulated Snail, Slug and c-Myc predominantly via the Smad2/3 pathway. Nevertheless, although the Smad2/3 pathway plays an important role in Nodal signaling, the results also displayed the complexity of this signaling pathway. Further studies should focus on the effect of non-Smad-associated pathways. We concluded that Nodal signaling induced EMT and up-regulated the expression of Slug, Snail and c-Myc via the Smad2/3 pathway, thereby facilitating VM formation.

We further verified the role of Nodal signaling in VM formation *in vivo*. In previous studies, Nodal expression increased tumorigencity and metastasis of glioma cells and breast cancer cells *in vivo* [19, 51, 52]. In this study, our findings also showed that Nodal promoted breast cancer cell tumorigencity and increased the tumor growth rate. Here, we demonstrated that Nodal expression promoted VM formation and increased the VM number in xenografts in a mouse model. This may provide one explanation for Nodal promotion of tumorigencity and metastasis. In a previous study, SB431542 was used to block Nodal signaling in B16 allografts, and inhibition of Nodal signaling *in vivo* reversed the EMT phenotype and inhibited metastasis [53]. In this study, we first used SB431542 to inhibit VM formation in human breast cancer xenografts. Importantly, it effectively reduced the VM number, thereby suppressing tumor growth. Consistent with the *in vitro* results, Nodal expression was positively correlated with the expression of VE-cadherin, MMP2 and Slug, while the expression of E-cadherin was reduced. Furthermore, SB431542-treated groups had lower VE-cadherin, MMP2 and Slug levels, and E-cadherin was up-regulated.

In conclusion, the clinicopathological evidence showed a correlation between the Nodal signaling pathway and malignant breast cancer progression. These results suggested that Nodal may be a diagnostic marker of poor prognosis. We demonstrated that Nodal signaling plays an essential role in promoting VM formation *in vitro* and *in vivo*. Although Nodal signaling involves complicated mechanism, the Smad2/3 pathway was shown to be important in VM formation. We conclude that Nodal up-regulates Slug, Snail and c-Myc expression via the Smad2/3 pathway, inducing EMT and thereby promoting VM formation. Furthermore, we examined the effects of blocking the Smad2/3 pathway *in vivo* and demonstrated that SB431542 applied to a breast cancer model could inhibit VM formation in xenografts. Consequently, the Nodal signaling pathway might serve as a therapeutic target for reducing VM formation, thereby improving the prognosis in breast cancer.

## MATERIALS AND METHODS

### Chemicals and reagents

The primary antibodies used in this study are listed in the supplementary material. The secondary antibodies were purchased from Zhongshan Golden Bridge Biotechnology Co., Ltd. (Beijing, China). SB431542 (S4317) was purchased from *Sigma*-Aldrich (St. Louis, MO), and SB431542 (S1067) was purchased from *Selleckchem* (Houston, TX). Matrigel was purchased from BD Biosciences (NY, USA).

### Patient samples

We obtained 100 random samples from patients who underwent surgical resection for breast cancer at the Tianjin Cancer Hospital of China from 1997 to 2005. The detailed pathological and clinical information of the patients was obtained. The median age of the patients was 48 years old (range, 27–74 years). The follow-up period was from the time of surgery to December 2008. The use of human specimens was approved by the Tianjin General Hospital Ethics Committee.

### Immunohistochemistry

IHC sections were pretreated in a microwave oven for 10 min at 95°C, blocked with goat serum, and incubated with antibodies overnight at 4°C (Supplementary Table S1). The staining systems PicTure PV6001 and PV6002 (Zhongshan Chemical Co., Beijing, China) were used. The slides were then incubated with DAB for 5–10 min and counterstained with hematoxylin. Negative controls were incubated with PBS instead of primary antibodies.

The positive staining in the breast cancer cells was assessed by two pathologists blinded to the patients' clinical pathology parameters using the staining index (SI) which was defined as described previously [54]. Five microscopic fields at 400×magnification were chosen randomly, and 100 tumor cells in each field were counted. In staining for Nodal, tumor cells with brown cytoplasm were considered positive and then scored based on four classes: none = 0; weak = 1; moderate = 2; and strong = 3. Percentage of stained tumor cells was categorized into four classes: 0 for negative cells, 1 ≤ 25%; 2 = 25–50%; 3 ≥ 50%. The sum (staining index) of intensity and percentage scores were utilized to determine the result. A staining index of ≥ 3 was defined as high expression, while < 3 was defined as low expression [55].

VM appeared as channels lined by tumor cells that were PAS-positive and CD31-negative, with red blood cells present but not endothelial cells (endomucin-stained endothelial cells in mouse tumors). After the CD31 (endomucin) IHC staining was performed, the slides were incubated first with 0.5% periodic acid solution for 15 min and then with PAS solution for 15–30 min in the dark and finally counterstained with hematoxylin. The number of VM was counted under 400× magnification, and the average number in five fields was recorded.

### Cell culture and treatment

The 293T cells and human breast cancer cell lines MDA-MB-231 and MCF-7 were cultured in DMEM medium with 10% FBS (Gibco) and 1% penicillin-streptomycin. The cell lines were purchased from the ATCC in 2012, and short tandem repeat (STR) analysis by Genewiz Inc. was used to confirm that the samples matched the reference cell lines in 2014.

Lentiviral expression plasmids with Nodal cDNA (catalog no. EX-T9592-Lv201) or a negative control (EX-NEG-Lv201) and lentiviral expression plasmids with Nodal shRNA (HSH011861-HIVU6) or a shRNA control (CSHCTR001-HIVU6) were purchased from GeneCopoeia, Inc. The lentivirus-mediated transfection was performed as described previously [56]. MDA-MB-231 cells were transfected with the Nodal shRNA vector (MDA-MB-231-shNodal) or the shRNA control vector (MDA-MB-231-shCon), and MCF-7 cells were transfected with the Nodal cDNA vector (MCF-7-Nodal) or the control (MCF-7-Con). Infected cells were selected with puromycin for 7 days.

For SB431542 (SB) treatment, MCF-7, MCF-7-Nodal and MDA-MB-231 cells were serum-deprived for 12 h and then treated with SB431542 (10 μM) for 48 h. The cells were collected for further assays.

### Western blotting

Whole cells were lysed with RIPA buffer. Protein lysates were separated on a 10% SDS-PAGE gel and electroblotted onto a PVDF membrane (Millipore). After the membrane was incubated with primary antibodies overnight, the secondary antibodies were added and incubated at room temperature (RT) for 2 h. After washing with TBS-Tween three times, an enhanced chemiluminescence detection kit was used. β-actin was used as a loading control, and the bands were assessed with a C-DiGit Blot Scanner (LI-COR) and analyzed using Image-Pro Plus.

### Polymerase chain reaction (PCR)

The details are provided in the Supplementary Materials.

### Gelatin zymography

The details are provided in the Supplementary Materials.

### Three-dimensional (3-D) cultures

Coverslips were coated with 20 μl Matrigel (BD) in 24-well plates. After 1 h at 37°C, the Matrigel transformed into gel, and breast cancer cells in complete DMEM were seeded onto the gel and were cultured with or without SB431542 at 37°C for 48 h. Capillary-like structure formation was filmed under a phase contrast microscope (200×).

For gel 3D cultures, after mixing with Matrigel, breast cancer cells were seeded into a 96-well plate. When gel was formed, complete DMEM was added. The other steps were the same as those described above.

### Immunofluorescence staining

The details are provided in the Supplementary Materials.

### Wound-healing and cell migration assays

A wound-healing assay was performed as described previously [6].

Migration assays were performed with an 8.0 μm pore filter chamber (Invitrogen) inserted in 24-well plates. The breast cancer cells (1 × 10^5^ cells) in 100 μL of DMEM without FBS were seeded into the upper wells, and DMEM and 10% FBS were added to the bottom chamber. The cells were incubated for 24–48 h. After fixing with methanol, the noninvading cells were removed from the upper surface. The invaded cells adhering to the bottom surface of the membrane were stained with 0.5% crystal violet. Using an inverted light microscope (Nikon), we counted the number of invading cells. All experiments were repeated independently at least three times.

### Cell invasion assay

The cells were seeded into the Matrigel-coated upper 24 wells (1 mg/mL; BD Biosciences), and the invasion assay was performed using the above protocol.

### Xenografts and treatments

Fifty-six female 4-week-old BALB/c-nu/nu mice were obtained from HFK Bioscience Co., Ltd. One week before the experiment, the mice were randomly divided into seven groups at the Animal Center of Tianjin Medical University (Tianjin, China). Cell (MDA-MB-231-shCon\MDA-MB-231-shNodal\MCF-7-Nodal\MCF- 7-Con) suspensions containing 5 × 10^6^ cells were subcutaneously injected into the upper right flank region at 0.1 mL/mouse. A stock solution (66 mg/ml) of SB431542 was prepared in dimethyl sulfoxide (DMSO). Three days after inoculation, the SB solution (10 mg/kg/mouse) was administered intraperitoneally to the treatment group on alternate days, and the control groups were intraperitoneally injected with buffer solution. Tumor volume was monitored weekly using vernier calipers and calculated using the following formula: TV = 1/2 × a × b^2^ (where a is the length and b is the width of the tumor). After 4 weeks, mice were sacrificed, and the xenograft tumors were removed, weighed, and processed for histology and immunohistochemical analysis.

### Statistical analysis

Analysis was performed using SPSS 21.0. The pathological and clinical characteristics of the two groups in breast cancer cases were assessed by the χ2 test. Mean values were assessed using a two-tailed Student's *t* test for paired data. Survival curves were estimated using the Kaplan-Meier method and compared by a log-rank test. Statistical significance was defined as *p* < 0.05.

## SUPPLEMENTARY MATERIALS


