# Supplementary material for: Nodal signaling promotes vasculogenic mimicry formation in breast cancer via the Smad2/3 pathway
Source: Oncotarget. 2016 Sep 21;7(43):70152–67. doi: 10.18632/oncotarget.12161 (PMC5342542; doi:10.18632/oncotarget.12161)
Supplement: Supplementary file 1 [file oncotarget-07-70152-s001.pdf]

Nodal signaling promotes vasculogenic mimicry formation in breast cancer via the Smad2/3 pathway

Supplementary Materials

Gelatin zymography

The serum-free media were separated on a 10% SDS-PAGE gel with 0.015% gelatin. After electrophoresis, the gels were equilibrated in 2.5% Triton X-100 and incubated in 50 mmol/L Tris-HCl (pH 7.5), 10 mmol/L CaCl2, 150 mmol/L NaCl, and 1 mmol/L ZnCl2 for 42 h at 37°C. The gels were then stained with 0.1% Coomassie Brilliant Blue and were destained until the bands associated with MMP activity became apparent.

RT-PCR

Total cellular RNA was extracted using TRIzol reagent (Invitrogen Life Technologies). cDNA was synthesized using QuantScript RT Kit (Tiangen Biotech). And GAPDH was used as loading control.
Primer sequences:
Nodal: Forward: 5′-GCGGTAGGCGTGTACGGT-3′
Reverse: 5′-CTGGAATAGCTCAGAGGC-3′
CDH5: Forward: 5′-AGCCAGCCCAGCCCTCAC-3′
Reverse: 5′-CCTGTCAGCCGACCGTCTTTG-3′
GAPDH: Forward: 5′-CCTGGCCAAGGTCATCC ATGAC-3′
Reverse: 5′-TGTCATACCAGGAAATGAGCTTG-3′

Immunofluorescence staining

Breast cancer cells on coverslips were washed once with PBS and fixed in absolute methanol for 20 min at –20°C, permeabilized with 0.1% Triton X-100 in PBS for 15 min at room temperature, and blocked for 30 min with 3% BSA in PBS. Then, the cells were incubated with primary antibodies overnight at 4°C. The cells were washed three times with PBS, followed by secondary antibodies conjugated with Alexa 488 or Alexa 568. DAPI (Sigma) was used to stain the nuclei, and the cells were examined using fluorescence confocal laser scanning microscopy (Nikon, Japan).

The rescue experiments had been performed by overexpressing Nodal in Nodal knockdown cells (shNodal4). Meanwhile, shNodal4 (more than 80%) and shNodal3 (70%) which had relatively effective knockdown effects (Supplementary Figure S2B) were chosen to performed the functional experiments. In Supplementary Figure S2A, MDA-MB-231 cells formed typical VM channels (red arrowhead). Meanwhile, when the expressions of Nodal in MDA-MB-231 cells were knocked down by shNodal4 and shNodal3, both MDA-MB-231-shNodal3 and MDA-MB-231-shNodal4 cells cannot form typical VM channels. However, Nodal knockdown cells (shNodal4) restored the VM channel forming ability when we rescued them by overexpressing Nodal again (Supplementary Figure S2 Ad). Moreover, the results showed that both shNodal3 and shNodal4 could inhibit migration and invasion abilities of MDA-MB-231 cells greatly. And we found that the migration and invasion abilities of Nodal knockdown cells (shNodal4) also got recovered in functional experiments when we performed rescue experiments to recover the Nodal expression (Supplementary Figur S2B, S2C).

Supplementary Table S1: Antibodies used in this study

Antibody	Source	IHC Concentration/ WB	Product Number	Manufacture
Nodal	Mouse	1:50 (IHC)	sc-81953	Santa Cruz
Nodal	Rabbit	1:1000 (WB)	ab109317	Abcam
Smad2/3	Rabbit	1:200 (WB)	sc-8332	Santa Cruz
p-Smad2/3	Goat	1:200 (WB)	sc-11769	Santa Cruz
VE-cadherin	Rabbit	1:500(WB) 1:400(IHC)	ab33168	Abcam
MMP2	Rabbit	1:200(WB) 1:100(IHC)	10373-2-AP	LuoSai-BIO
MMP9	Rabbit	1:500(WB)	ab76003	Abcam
E-cadherin	Mouse	1:200(WB) 1:100(IHC)	ab1416	Abcam
N-cadherin	Rabbit	1:1000(WB)	AF4039	Affinity Biosciences
Vimentin	Rabbit	1:500(WB) 1:200(IHC)	ab92547	Abcam
Slug	Rabbit	1:200(WB)<br>1:100(IHC)	ab27568	Abcam
Snail	Rabbit	1:200(WB)	ab180714	Abcam
c-Myc	Rabbit	1:500(WB)	LS-137576	LSBio
β-actin	Rabbit	1:2000(WB)	P30002	Abmart
CD31	Rabbit	1:100(IHC)	ZM-0044	ZSGB-BIO
Endomucin	Rat	1:800(IHC)	14-5851-81	Ebioscience

Note: IHC: Immunohistochemistry, WB:Western blot.

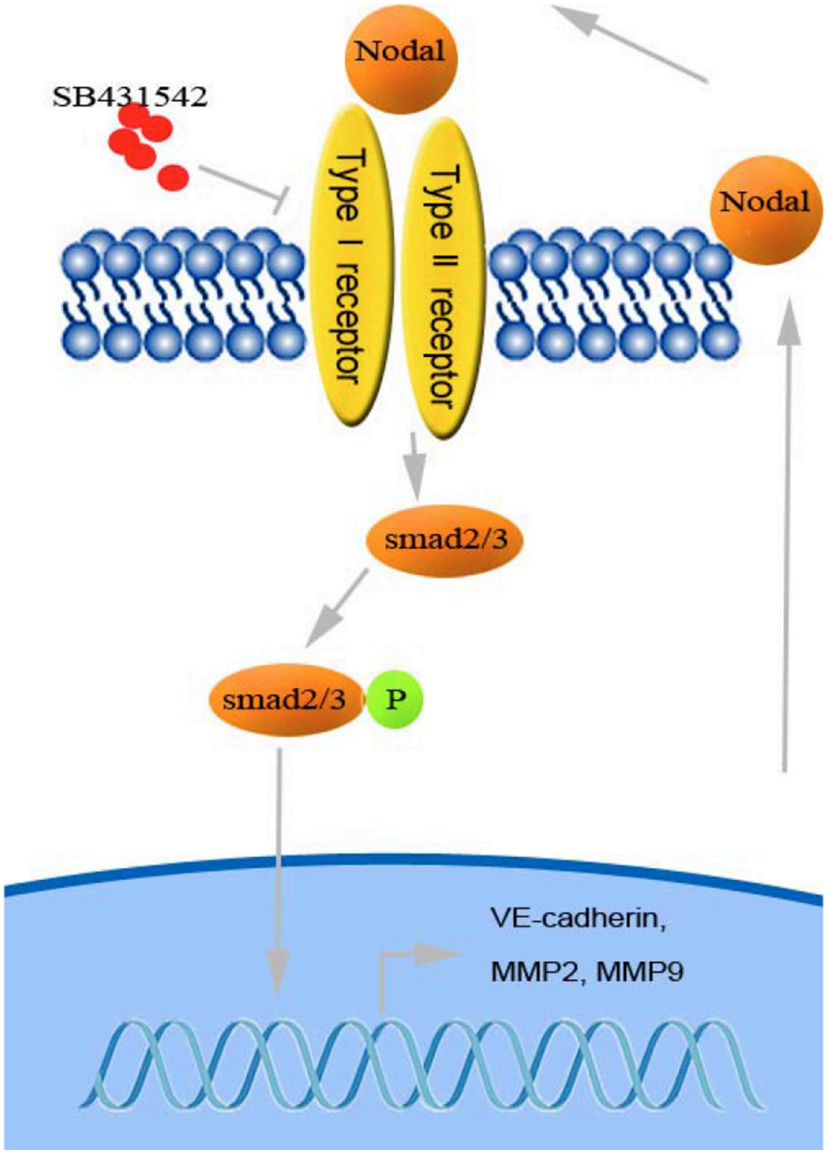

Supplementary Figure S1: Nodal is a secreted protein. It binds to activin-like kinase type II and type I receptors, which leads to phosphorylation of Smad2/3 and regulates target genes. Even if the Nodal expression was up-regulated, the inhibition of activin receptors by SB431542 prevented Nodal working through this pathway, resulted in expression of p-smad2/3, VE-Cadherin, MMP2 and MMP9 not reversing.

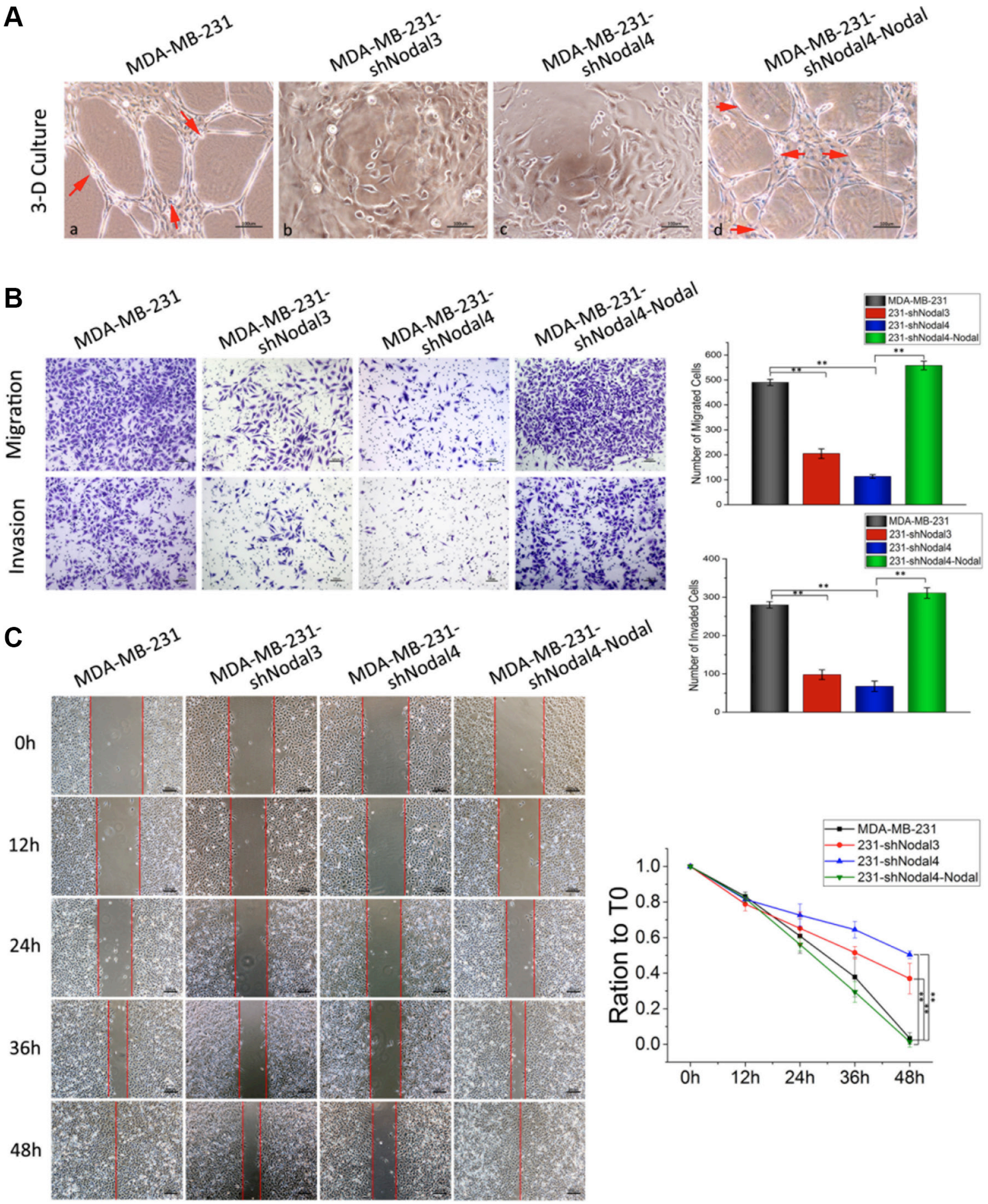

Supplementary Figure S2: Functional experiments in MDA-MB-231, MDA-MB-231-shNodal3, MDA-MB-231-shNodal4 and MDA-MB-231-shNodal4-Nodal cell lines. (**A**) MDA-MB-231 cells formed typical VM channels (red arrowhead) (Supplementary Figure S2 A a). MDA-MB-231-shNodal3 and MDA-MB-231-shNodal4 cells cannot form typical VM channels. However, Nodal knockdown cells restored the VM channel forming ability when we rescued them by overexpressing Nodal again (red arrowhead) (Supplementary Figue S2 A d). (**B**) transwell assays were performed in the indicated groups. Scale bar = 100 μm. (**C**) wound-healing assays were performed to verify the migrated abilities in the indicated groups. Scale bar = 200 μm. The data are presented as the mean ± standard deviation (SD). ***p* < 0.01.
